# Municipality-level characteristics associated with very good quality water fluoridation in Brazil in 2018

**DOI:** 10.1590/1807-3107bor-2024.vol38.0082

**Published:** 2024-12-09

**Authors:** Lorrayne Belotti, Camila de Moraes Paulino, Paulo Frazão

**Affiliations:** a Hospital Israelita Albert Einstein, Albert Einstein Center for Studies, Research, and Practices in Primary Health Care and Networks, Sao Paulo, SP, Brazil.; b Universidade de São Paulo – USP, School of Public Health, Department of Politics, Management and Health, São Paulo, SP, Brazil.

**Keywords:** Fluoridation, Water Supply, Oral Health, Public Health

## Abstract

The aim of this study was to analyze factors associated with the quality of fluoridation in water supply systems in Brazil in 2018. An ecological study was conducted using official data sources on fluoridation provision and fluoride concentration surveillance in 2018. Inclusion criteria were municipalities with a water supply system and accurate data. Municipalities meeting the quality standard, defined as having 80% or more of water samples within the optimal level for caries prevention were classified as "very good" fluoridation quality. Independent variables encompassed structural aspects, socioeconomic factors, and indicators of managerial and technical capabilities in water surveillance. Prevalence ratios were estimated using Poisson regression with robust variance. A total of 58.9% of municipalities with water supply systems had fluoride-adjusted water, with 65.3% adequately contributing to the surveillance data system. Among these, 42.2% exhibited "very good" fluoridation quality. Quality was higher in larger municipalities with elevated socioeconomic indexes and per capita expenditure on sanitary surveillance above the median. Additionally, municipalities with a conformity rate of free residual chlorine concentration of 75% or higher had better fluoridation quality. After adjusting for all variables, fluoridation quality was lower in municipalities with above-median per capita gross domestic product, higher socioeconomic inequality, and below-median monthly household per capita income. Fluoridation quality was linked to municipality-level characteristics concerning structural aspects, socioeconomic factors, and managerial and technical factors in water surveillance. The information generated regarding the coverage features of surveillance and fluoridation strategies can be highly valuable for redirecting inter-sectoral public policy management.

## Introduction

Several nations around the world regulate the fluoride levels in drinking water to prevent dental caries in the population. Community water fluoridation (CWF) is not only cost-effective with substantial preventive benefits, but also addresses social inequalities, ensuring access to fluoride for diverse socioeconomic strata within the population connected to the public water supply.^1^ In countries such as Australia, Brazil, Canada, the United Kingdom, and the United States (US), there are still disparities among the geographic regions benefited by water fluoridation, despite its recognized importance as a public health strategy. To maximize the effectiveness of dental caries prevention through water fluoridation, it is crucial that optimal fluoride levels are maintained over time. The ability to maintain this standard is considered a hallmark of a high-quality CWF.^2,3^ Despite technological advances in systems to regulate fluoride concentration, a high variation has been found in different water supply systems.^4–6^ One of the recommendations approved at the 74th World Health Assembly is the mapping and testing of fluoride concentration in drinking water.^7^

The primary strategy for ensuring water quality is surveillance,^8^ and fluoridation quality depends on this surveillance framework.^9^ The establishment of this framework relies on various determinants, including an integrated regulatory system comprised of different organizations, each with a specific purpose within the entire chain from watershed to tap, and these organizations are designed to verify the efficacy of water safety plans.^10^ In countries with governmental structures and political processes based on federalist systems, the integration of organizations responsible for water quality largely relies on subnational units, whether at the state or municipal level. The implementation of policies in the fluoridation strategy, often linked to the sanitation sector, can differ from that in the surveillance strategy, associated with the health sector. These sectors, subject to distinct interests and with their own political dynamics, may undergo varied levels of policy implementation.

Economic indicators, such as annual Gross Domestic Product per capita (GDP),^11^ human development level,^12^ and income inequality^13^ measured at the municipal level have been associated with dental caries and could be linked with the quality of CWF. Although the health status of the population is crucial for economic growth, it's important to note that economic development doesn't necessarily lead to overall health improvement or reduction of health disparities. Wealthier and larger cities often have better infrastructure, enabling them to implement more effective public policies.^14^ It's noteworthy that the relationship between a robust economy and health tends to weaken once annual GDP per capita surpasses a threshold of US$ 5,000.^15^

Presently, there is limited information on the correlation between sustaining an optimal fluoride level over time and municipality-level characteristics associated with sanitation, health, and economic variables, despite evidence supporting a connection between implementation of public policies and territorial features.^16^

During the 1980s, there was an expansion of water fluoridation in Brazil, facilitated by the initiation of a government program that provided credit and technical assistance to public water treatment companies. Data from 2008 revealed variations in the provision of CWF in Brazilian municipalities based on population size and Human Development Index (HDI).^17^ Following the expansion, reports from specific areas indicated that the maintenance of fluoridation did not conform to the established standard. These reports led to the development of surveillance systems based on external controls that focused on assessing the adequacy of fluoride content, using water samples from various points within the supply network.^2^ Since 2000, the municipal health authorities have assumed responsibility for conducting surveillance actions and implementing their own water sampling plans. Furthermore, since 2007, municipalities have been authorized to conclude contracts with public or private sanitation companies.^18^

However, there hasn't been a nationwide study examining municipality-level characteristics related to fluoridation quality. Two regional studies have indicated connections with factors such as population size, human development level, infant mortality, per capita household income, and chlorine concentration conformity rate.^4,16^ Investigating the interactions between these factors could offer scientific insights to improve the intersectoral management of health and sanitation. In light of these considerations, the objective of this study was to analyze factors associated with the quality of fluoridation in water supply systems in Brazil in 2018.

## Methods

An ecological study based on water surveillance official data was carried out covering the municipalities in Brazil in 2018.

### Study area

Brazil is governed by a three-level federal system that assures power and relative autonomy for the central government (first level), 26 states and one federal district (second level), and 5,570 cities (third level). In 2018 there were about 208.5 million inhabitants and two out of three lived in municipalities with 50 thousand or more inhabitants. Although the country is part of the group of upper-middle income countries and has experienced growth and reduction of inequalities at the beginning of the 21st century, its ranking of social indicators is still far behind its position in the ranking of economic indicators: in 2020 Brazil ranked 12th in gross national income and 81st in life expectancy at birth.^19^ The Unified Health System, institutionalized in 1988 under the influence of a broad health reform movement, created the conditions to ensure an important share of power and autonomy to sub-national governments, favoring political-administrative decentralization of health actions, including surveillance actions. Official data from the National Basic Sanitation Research undertaken in 2017 showed that 86.1% of the population had access to treated water. Since 2000, state and municipal authorities have been responsible for water surveillance and must monitor compliance with the safety and quality standards of drinking water supplied by the companies. Therefore, all water supplied to the population through water supply systems and alternative supply solutions must comply with the physical, chemical, and microbiological requirements defined by the current legislation, so that it does not pose a risk to users. Adjusting the fluoride concentration for dental caries prevention is mandatory in water supply systems where there is a water treatment plant. Out of 5,570 Brazilian municipalities, 4,659 were supplied with water by public systems, and 4,442 had information on fluoridation (yes or no) in the National Water and Sanitation Data System.^20^

### Data source

We used official data on fluoride concentration in the water supply systems of the Brazilian municipalities registered in the Drinking Water Quality Surveillance Information System in 2018. Municipalities that did not feed the information system for at least four months were not included in the analysis. Verification procedures followed a protocol for data assessment published elsewhere.^21^

### Dependent variable

Fluoridation quality was determined by the percentage of fluoride concentration values falling within the optimal range in each municipality. This range corresponds to the maximum benefit in preventing dental caries while minimizing the risk of dental fluorosis, as outlined in the Technical Consensus on Classification of Public Water Supply in Relation to Fluoride Content endorsed by CECOL/USP (Equation 1).


$$Fluoridationquality=\frac{N^{\circ }ofsuitablesamples\;\left(0.55\leq T_{fucrido}\leq 0.94\right)}{Totalnumberofsamples\;(n)}$$

After calculating the proportion of samples within the optimal range, the fluoridation quality in municipalities was assessed using a compliance criterion in which municipalities with 80% or more of their samples meeting the standard were categorized as having "very good" fluoridation quality, while those with less than 80% were categorized as "other".^3^

### Independent variables

Chart 1 describes the explanatory variables and respective categories, year, and data source.

### Data analysis

To identify municipal characteristics associated with the optimal fluoride level in drinking water supply, the reference category for the outcome was set as the "very good" quality of fluoridation. Given the prevalence of the outcome and its overdispersion, Poisson regression analysis with robust variance was performed to estimate both crude and adjusted prevalence ratios (PR) along with their respective 95% confidence intervals. To check collinearity, independent variables with possible similarity were analyzed in specific blocks. Values of the variance inflation factor (VIF) and tolerance (1/VIF) were also examined. All explanatory variables with p values lower than 0.20 were included in the specific blocks and p values lower than 0.05 were included in the final model.

**Chart 1:** Summary of the independent variables included in the study.

Variable	Description	Categorization	Year	Source^a^
Population Size	Inhabitants residing in the municipalities under analysis	10,000	2018	IBGE
		10,000 up to < 50,000		
		50,000 or more		
Per capita Gross Domestic Product in Reals (GDP) *	Sum of all monetary value of final goods and services produced by the municipality divided by the population of each municipality.	≤ 27,005	2018	IBGE
		> 27,005		
Human Development Index (HDI)	Geometric mean of normalized indices on 3 dimensions: health (life expectancy at birth), education (mean years of schooling for adults, 25+ y), and standard of living (gross national income per capita) transformed to a scale from 0 (lowest) to 1 (highest).	< 0.600 (Very low and low)	2010	IBGE
		0.600 a 0.699 (Medium)		
		≥ 0.700 (High and very high)		
Firjan Municipal Development Index (FMDI)*	A measure of strength and quality of the formal labor market (includes aspects of formal employment generation and rate, wage bill and inequality); quality of education (includes indicators of enrollment, dropout, age-grade distortion, daily class hours, value of the basic education development index; quality of health (includes number of prenatal consultations per pregnant woman, deaths from uncertain causes, infant deaths from preventable causes and hospitalization sensitive to primary care)	≤ 0.74	2016	FIRJAN
		> 0.74		
Per capita monthly household income in Reals*	Ratio between the sum of income of all individuals residing in permanent private households and the total number of individuals	≤ 645.25	2010	IBGE
		> 645.25		
Gini Index*	Indicator of social inequality. It points out the difference between the income of the poorest and the richest. Varies from zero (no inequality) to one (maximum inequality)	≤ 0.46	2010	IBGE
		> 0.46		
Per capita expenditure on sanitation and health surveillance*	Expenditure on sanitary surveillance per inhabitant in 2018, an indicator of municipal management performance	≤ 13.09	2018	SICONFI
		> 13.09		
Conformity rate for free residual chlorine concentration *	Relative frequency of total samples with values between 0.2 mg/L and 2.0 mg/L in 2018	< 75%	2018	SISAGUA
		≥ 75%		

* Variables categorized by the median; IBGE: Brazilian Institute of Geography and Statistics; SISAGUA: Information System for Surveillance of the Quality of Water for Human Consumption; FIRJAN: Federation of Industries of the State of Rio de Janeiro; SICONFI: Information System for Accounting and Fiscal Data of the Brazilian Public Sector.

The hypothesis tested was based on the premise that the effect of certain municipality-level characteristics of policy provision could differ from the observed effect on fluoridation quality, and that such characteristics could interact with each other and be directly related to the outcome. First, we present the scenario of water fluoridation provision. Next, we examined fluoridation quality based on data surveillance. The analysis was conducted in three blocks according to the conceptual framework shown in Figure. Variables related to structural aspects and socioeconomic factors such as population size, per capita annual GDP, municipal Human Development Index (MHDI), and Firjan Municipal Development Index (FMDI) were included in block 1 and variables related to households such as per capita monthly household income and Gini index were included in block 2. Indicators related to managerial and technical aspects of water surveillance such as per capita expenditure on health surveillance and conformity rate for free residual chlorine concentration were grouped in block 3.

**Figure:**
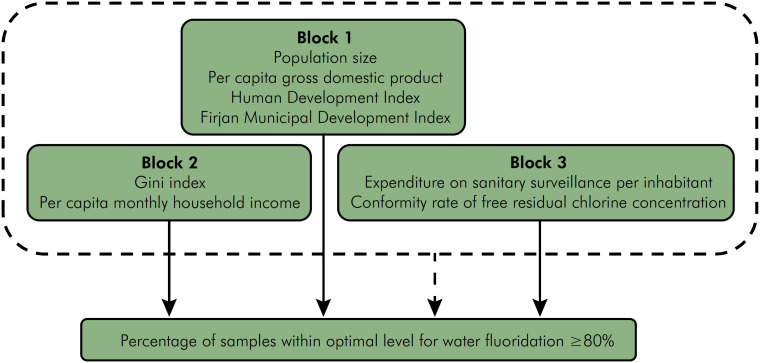
Analytical model of variables included.

Population size is an indication of the attractiveness of the place throughout its history, probably as a result of long-standing public policies that allowed the growth of diverse economic sectors. It has shown a strong positive correlation with fluoridation quality, according to earlier studies.^4,16^ Brazilian municipalities have marked differences in per capita, GDP, MHDI, and FMDI. GDP is an indicator of the total economic value of all the goods and services produced within that municipality's borders during a year. MHDI and FMDI measure similar domains by combining different indicators. While the former combines dimensions of life expectancy at birth, mean years of schooling for adults (25+y), and gross national income per capita, the latter includes aspects of formal employment generation and employment rate, wage bill and inequality, indicators of school enrollment and dropout, age-grade distortions, daily hours of schooling, the value of the basic education development index, and the number of prenatal consultations per pregnant woman, deaths from undetermined causes, infant deaths from preventable causes, and hospitalization that respond to primary care. Both indices are based on official data and used to monitor local socioeconomic development, but the time lag of FMDI is lower, only two years, and it covers a larger number of variables. Local socioeconomic development has a clear correlation with sanitation conditions,^22,23^ and lower hospitalization rates due to diarrhea can be associated with water quality,^24^ so some impact on water fluoridation can be expected.

The living standard per household is an important factor to explain the general health status of a population until a determined level^8^. Per capita income is related to access to material resources relevant to health in most societies, including clean water, sanitation facilities, adequate nutrition, and housing. Differences related to monthly per capita household income and the Gini index included in block 2 could be associated with sanitation and health policies, such as water fluoridation and treatment. Income inequality has been associated with underinvestment in public policies, particularly education and health care.^25,26^ A study in a major Brazilian state showed that, when adjusted by the Gini index, the prevalence of municipalities with less than 80% of water samples within the optimal level for caries prevention was 32% higher in those with a lower annual GDP per capita.^4^

Finally, the per capita expenditure on health surveillance and the conformity rate of free residual chlorine concentration are the most directly related variables to the managerial and technical factors of water surveillance. The compliance rate of chlorine concentration is an important indicator of treated water quality,^8^ and a direct association with fluoridation quality could be expected.^4^

We presented the effect of adjusting the model by variables in each block on the outcome to assess how the variables interact with each other and their overall effect in order to verify which maintained a significant association in the presence of the remaining ones (Figure). The analyses were performed using the Stata 14.1 program (Stata Corporation, 2013, College Station, USA).

## Results

Out of 4,442 Brazilian municipalities with official data, 2,746 had sanitation utilities that reported adjusting fluoride concentration for dental caries prevention. The number of municipalities that collected water samples and had fluoride values registered in the information system was even smaller. Table 1 presents the implementation of surveillance and fluoridation strategies. It is worth noting that 58.9% of municipalities with water supply systems had fluoride-adjusted water, of which 65.3% adequately fed the surveillance data system.

**Table 1:** Implementation scale of fluoridation and surveillance strategies in Brazil in 2018.

Municipalities	n (%)	%
Total	5,570 (100)	
Served by water supply system	4,659 (83.6)	
Served by fluoride-adjusted water	2,746 (49.3)	58.9^a^
Adequate Feeding of the Surveillance System	1,794 (32.2)	65.3^b^

a Percent municipalities served by water supply system;

b Percent municipalities served by fluoride-adjusted water.

Table 2 shows the characteristics of municipalities with and without fluoridated water according to independent variables. The scenario reveals that the rate of benefit from fluoridated water was higher in municipalities with lower Gini index, with higher values of per capita gross domestic product, HDI, FMDI, monthly per capita household income, per capita expenditure on sanitary surveillance, and conformity rate of free residual chlorine concentration. Of municipalities that declared adjusting fluoride concentration, 1,794 (65.3%) had fluoride values in the surveillance system that met the data assessment protocol. Out of them, 757 (42.2%) had "very good" fluoridation quality.

**Table 2:** Percentage distribution of municipalities in Brazil in 2018 served by water supply systems according to the provision of fluoridation and independent variables. (n = 4,442).

Variable	Category	Provision of fluoridation	
		n = 1,696	n = 2,746
		No (%)	Yes (%)
Population size	< 10,000	34.9	65.1
	10,000 to < 50,000	43.0	57.0
	50,000 or more	32.7	67.3
Per capita gross domestic product* (median)	≤ 19,200	56.1	43.9
	> 19,200	20.3	79.7
Municipal Human Development Index	< 0.600	84.4	15.6
	0.600 to 0.699	42.4	57.6
	0.700 or more	12.8	87.2
FMDI^a^*	≤ 0.69	57.7	42.3
	> 0.69	19.4	80.6
Gini Index*	≤ 0.49	24.3	75.7
	> 0.49	51.7	48.3
Per capita monthly household income*	≤ 507.76	62.2	37.8
	> 507.76	15.4	84.6
Per capita expenditure on sanitary surveillance*	≤ 9.47	43.8	56.2
	> 9.47	32.6	67.4
Conformity rate of free residual chlorine concentration*	< 75%	47.7	52.3
	≥ 75%	30.2	69.8

a FMDI: Firjan Municipal Development Index.

Table 3 shows the number and percentage of municipalities in the categories "Very Good" and other. "Very good" fluoridation quality was more frequent in cities with a population greater than 50,000 inhabitants, with highest per capita gross domestic product, with an MHDI greater than 0.700, and with the FMDI greater than 0.74. Furthermore, municipalities with a Gini index below 0.46, per capita income higher than R$ 645.25, greater per capita expenditure on sanitary surveillance, and a higher conformity rate of free residual chlorine concentration showed a higher prevalence of "very good" quality of fluoridation.

**Table 3:** Percentage distribution of municipalities in Brazil in 2018 according to the quality of fluoridation and independent variables.

Variable	Category	Fluoridation		Total	p-value^a^
		Other categories	Very good	n (%)	
		n (%)	n (%)		
Population size	< 10,000	470 (60.6)	306 (39.4)	776	< 0.001
	10,000 to < 50,000	434 (61.0)	278 (39.0)	712	
	50,000 or more	133 (43.5)	173 (56.5)	306	
Per capita gross domestic product* (median)	≤ 27,005	517 (57.6)	380 (42.4)	897	0.886
	> 27,005	520 (58.0)	377 (42.0)	897	
Human Development index	< 0.600	48 (92.3)	4 (7.7)	52	**< 0.001**
	0.600 to 0.699	370 (73.7)	132 (26.3)	502	
	0.700 or more	616 (49.8)	621 (50.2)	1237	
FMDI^b^ *	≤ 0.74	600 (66.9)	297 (33.1)	897	**< 0.001**
	0.74	437 (48.7)	460 (51.3)	897	
Gini Index*	≤ 0.46	488 (52.8)	436 (47.2)	924	**< 0.001**
	0.46	549 (63.1)	321 (36.9)	870	
Per capita monthly household income*	≤ 645.25	554 (61.8)	342 (38.2)	896	**0.001**
	645.25	483 (53.8)	415 (46.2)	898	
Per capita expenditure on sanitary surveillance*	≤ 13.09	555 (61.9)	342 (38.1)	897	**< 0.001**
	> 13.09	482 (53.7)	415 (46.3)	897	
Conformity rate of free residual chlorine concentration*	< 75%	281 (74.5)	96 (25.5)	377	**< 0.001**
	≥ 75%	756 (53.4)	661 (46.6)	1417	

* Median;

a Chi-square test;

b IFDM: Firjan Municipal Development Index.

Table 4 presents the associations between municipal characteristics and the outcome, which is defined as 80% or more of samples within optimal fluoride level range. The second column shows the adjusted values in each block. In block 1, per capita GDP was not included because its p-value was > 0.20. In the final model, MHDI was removed because of multicollinearity (VIF > 10), and per capita monthly household income was removed because of a p-value > 0.05. All remaining variables were significant and the effect of population size on the outcome increased by controlling for other variables. Municipalities with 50 thousand or more inhabitants had a 1.55 times higher prevalence of the outcome compared to smaller municipalities (95%CI 1.34–1.77). The magnitude of prevalence ratio values reduced slightly but remained significant for other variables. The fluoridation quality was higher in municipalities with values above the median FMDI (PR = 1.41, 95%CI 1.26–1.57) and the median per capita expenditure on sanitary surveillance (PR=1.15, 95%CI 1.04–1.28), and in those with a conformity rate of free residual chlorine concentration 75% or higher (PR = 1.69, 95%CI 1.17–1.26) compared to their counterparts. On the other hand, the fluoridation quality was lower in municipalities with higher socioeconomic inequality (Gini index PR = 0.77, 95%CI 0.69–0.86).

**Table 4:** Crude and adjusted analysis of municipal characteristics in Brazil in 2018 associated with more than 80% of fluoride samples in the optimal level range for prevention of dental caries.

Variable		Category	Non-adjusted Values			Adjusted values in each Block			Final Model		
			PR	95%CI	p-value	PR	95%CI	p-value	PR	95%CI	p-value
Block 1											
	Population size	<10,000	1.00	–	–	1.00	–	–	1.00	–	–
		10,000 to < 50,000	0.99	0.87–1.12	0.878	1.01	0.89–1.14	0.806	1.06	0.94–1.20	0.317
		50,000 or more	1.43	1.26–1.64	< 0.001	1.27	1.12–1.44	< 0.001	1.55	1.34–1.77	< 0.001
	Per capita gross domestic product*	≤ 27,005	1.00	–	–		Not	–		Not	–
		> 27,005	0.99	0.89–1.11	0.886		included			included	
	Human Development index	< 0.600	1.00	–	–	1.00	–	–			
		0.600 to 0.699	3.48	1.32–8.87	0.011	3.22	1.23–8.43	0.017		withdrawn^c^	
		0.700 or more	6.53	2.54–16.76	< 0.001	5.48	2.11–14.21	< 0.001			
	FMDI^b^ *	≤ 0.74	1.00	–	–	1.00	–	–	1.00	–	–
		> 0.74	1.55	1.38–1.73	< 0.001	1.31	1.17–1.47	< 0.001	1.41	1.26–1.57	< 0.001
Block 2											
	Gini index*	≤ 0.46	1.00	–	–	1.00			1.00	–	–
		> 0.46	0.78	0.70–0.87	< 0.001	0.78	0.70–0.87	< 0.001	0.77	0.69–0.86	< 0.001
	Per capita monthly household income*	≤ 645.25	1.00	–	–	1.00	–	–			
		> 645.25	1.21	1.08–1.35	0.001	1.21	1.08–1.35	0.001		withdrawn^d^	
Block 3											
	Per capita expenditure on sanitary surveillance*	≤ 13.09	1.00	–	–	1.00	–	–	1.00	–	–
		> 13.09	1.21	1.09–1.35	0.001	1.18	1.06–1.31	0.003	1.15	1.04–1.28	0.009
	Conformity rate of free residual chlorine concentration*	< 75%	1.00	–	–	1.00	–	–	1.00		
		≥ 75%	1.83	1.53–2.20	< 0.001	1.81	1.51–2.17	< 0.001	1.69	1.17–1.26	< 0.001

* Median;

a Wald test;

b IFDM: Firjan Municipal Development Index;

c Variance Inflation Factor >10;

d p-value > 0.05.

## Discussion

Based on 2018 official data on water surveillance produced by Brazilian municipal health authorities, 42.2% of included municipalities had 80% or more fluoride samples in the optimal interval for caries prevention, a value slightly lower than that observed in a study conducted in a Brazilian sub-national territory^4^. A longitudinal analysis in New Zealand indicated that fluoride data was available for 82% of people on a fluoridated supply, and water suppliers achieved fluoride targets (0.7 to 1.0 mgF/L) 54.1% of the time overall.^27^ A similar study in England revealed that 71.1% of fluoride-adjusted areas had values within expected average concentrations between 2009 and 2020.^28^

The results showed, one step at a time, that the fluoridation policy reached 49.3% of Brazilian municipalities and that surveillance was adequate in 65.3% of those in which fluoride concentration in the drinking water was adjusted for dental caries prevention by sanitation companies. These data are a good indication of three points: the stage of implementation the municipalities are at; the existing space for expansion of each of them; and that surveillance and fluoridation strategies have their own dynamics, even if they are subject to common economic and political conditions. Analysis of fluoride use in Brazil show major differences in coverage and trajectory according to the strategy: systemic use by water fluoridation and topical use (from direct application of solutions to fluoride toothpaste). From 1952 to 2017, the first strategy, which was dependent on the sanitation sector, experienced an progressive increase, while the second strategy, conducted by the health sector, presented an increase followed by stagnation and a decline, drawing an inverted parabola.^18^

Municipality-level variables related to structural aspects, socioeconomic dimensions, and managerial and technical factors related to water surveillance showed an independent association with fluoridation quality. To the best of our knowledge, this is the first nationwide ecological study determining how these factors interact with each other. Structural factors such as population of 50,000 or more and composite indicators such as MHDI and FMDI were associated with higher levels of fluoridation quality. It is important to highlight that MHDI was removed from the final model because of a possible collinearity with other independent variables that inflate the standard errors of the coefficients. Studies in two Brazilian sub-national territories found that very good quality of CWF was also higher in municipalities with larger populations and higher HDI.^4,16^ Investigations have shown that cities with more robust economies have higher tax revenue and more appropriate structural conditions to provide better public policies.^14^ The higher quality levels observed in municipalities with larger populations can be attributed to more favorable municipal conditions as a result of long-standing public policies, which favor the growth of diverse economic sectors, leading to better infrastructure, organizational learning, human resources, and extensive technical and business expertise.^29^ In smaller Brazilian municipalities, maintaining adequate fluoride concentrations in public water supply may be problematic due to limited availability of specialized workforce, inadequate training for water treatment plant operators, insufficient inspection infrastructure, and limited experience in controlling the fluoridation process.^30^

In a qualitative investigation in small Brazilian towns, researchers found that institutional characteristics such as administrative weakness of local entities, the low priority given to policy at the local level, the poor physical structure of water treatment plants, isolated working relations, the low effectiveness of monitoring devices, and the uncertainties of local actors about the policy favored the expansion of street-level operators with discretionary power, creating important barriers for water fluoridation.^31^

Higher income inequality was independently associated with fewer fluoride samples in the optimal interval for caries prevention. This association is compatible with the notion that an area-level income inequality would reflect in lack of material resources and systematic underinvestment in social infrastructure such as public policies on housing, sanitation, transportation, healthcare, and welfare.^32^ It is worth noting that higher per capita monthly household income was positively associated with very good fluoridation quality in the univariate analysis and in the analysis adjusted by income inequality. A study comprising 645 Brazilian municipalities also found this positive association.^4^ This finding is in line with the observed dependency, in which municipalities with higher monthly per capita household income also had lower income inequality. A thorough analysis based on data from the 1995–2015 Monthly Employment Surveys conducted by the Brazilian Institute of Geography and Statistics in approximately 40 thousand housing units located in the country's main metropolitan areas showed that income gains in the different strata correlated negatively with income inequality.^33^ However, monthly per capita household income lost significance in the final model, showing that the outcome is dependent on structural factors and socioeconomic dimensions such as population size, FMDI, and Gini index.

The current study also found a positive association between fluoridation quality and managerial and technical factors of the municipalities. The better the control of chlorine in public water supply, the better the fluoridation quality independently of other variables. Higher fluoridation quality was associated to above-median per capita expenditure on health surveillance, which is an economic-fiscal factor that expresses the extent to which the municipality is able to incur expenditure related to environmental surveillance activities, notably those related to water surveillance.^34^

The results reported in this study were based on official data that revealed distinctive implementation scales of surveillance and fluoridation strategies. One study limitation is the lack of information on the characteristics of sanitation companies. Earlier studies have shown that such characteristics can be associated with both strategies.^4,35^ Another limitation is the lack of variables related to operations of water treatment undertaken by sanitation companies and water surveillance operations carried out by the local health systems. In an integrated regulatory system aimed at ensuring safe water, the characteristics related to this organizational level could be important. Some studies have described significant variation in the level of knowledge about fluoridation of water plant operators^36^ and of water surveillance workers,^37^ which might reflect weak levels of structuring and institutionality of certain practices within local organizations. Despite this, the study showed characteristics related to the municipality, a strategic entity for building an integrated regulatory system directed to ensure safe drinking water.

In conclusion, the quality of fluoridation was associated with municipality-level structural aspects, socioeconomic dimensions, and indicators of managerial and technical capabilities related to water surveillance. The information produced on the characteristics of surveillance and fluoridation strategies can be extremely useful for refocusing inter-sectoral of public policy management.
